# Plasma zinc, copper and serum ceruloplasmin levels of autism spectrum disorder children in Bangladesh

**DOI:** 10.1016/j.heliyon.2023.e18624

**Published:** 2023-08-09

**Authors:** Umme Raihan Siddiqi, Shelina Begum, Shorifa Shahjadi, Sharmin Afroz, Syeda Nusrat Mahruba, Jobaida Parvin, Md Mahbubur Rahman

**Affiliations:** a Department of Physiology, Mymensingh Medical College, Mymensingh, Bangladesh; b Department of Physiology, Bangabandhu Sheikh Mujib Medical University, Shahbag, Dhaka, Bangladesh; c Department of Physiology, Ibrahim Medical College, Shahbag, Dhaka, Bangladesh; d Department of Pediatric Neurology, National Institute of Neurosciences and Hospital, Sher-E-Bangla Nagar, Dhaka, Bangladesh; e Department of Computer Science and Engineering, Military Institute of Science and Technology, Mirpur Cantonment, Dhaka, Bangladesh

**Keywords:** Autism spectrum disorder (ASD), Zinc (Zn), Copper (Cu), Zinc/copper ratio (Zn/Cu ratio), Ceruloplasmin (Cp)

## Abstract

Neural and cognitive processes require zinc and copper homeostasis and a normal zinc/copper ratio. Ceruloplasmin, an intrinsic antioxidant protein, maintains copper homeostasis, which might also influence autism spectrum disorder (ASD). ASD children are frequently reported with altered levels of these elements with wide geographical variations. This study evaluated any alteration in plasma zinc, copper, zinc/copper ratio and serum ceruloplasmin levels in Bangladeshi ASD children with respect to healthy controls. A cross-sectional study was conducted on 67 children aged 2 to 9 years of both sexes. Among them, 35 had ASD, while 32 were age, sex and body mass index (BMI) matched apparently healthy children. Plasma zinc and copper levels were estimated by the flame atomic absorption spectrophotometry method. Serum ceruloplasmin levels were estimated by the immunoturbidimetric method. Zinc and zinc/copper ratio in the 2–9 years old ASD children group were significantly lower (p=0.032 and p=0.002 respectively). On the other hand, copper (p=0.020) and ceruloplasmin (p = 0.045) levels were significantly higher than those of apparently healthy children. ASD was significantly associated with zinc deficiency (p=0.000) and copper toxicity (p=0.05). All children were again divided into 2–5 and 6–9 years age groups according to laboratory reference values for zinc and copper. Copper toxicity was significantly associated with ASD in the 2–5 years old age group (p=0.011), with a significant difference in plasma copper levels (p=0.009) and zinc/copper ratio (p=0.001) but not serum ceruloplasmin levels (p=0.110) compared to healthy controls. Serum ceruloplasmin was positively associated with plasma copper in ASD children of all age groups. This study shows that ASD in Bangladesh can be associated with low plasma zinc and high plasma copper and serum ceruloplasmin levels.

## 1 Introduction

Autism spectrum disorder (ASD) is a complex neurodevelopmental disorder having persistent impairment in communication and social interaction, along with restrictive and repetitive behaviour, interests and activities [1]. ASD includes autistic disorder, Asperger syndrome and pervasive developmental delay not otherwise specified, can appear at any time during the neurodevelopmental period [2,3]. In the last few decades, the incidence of ASD has increased globally as well as in Bangladesh at an alarming rate [4,5]. The interaction among environmental and biological factors may result in epigenetic alterations of neurodevelopmental regulatory mechanisms [[6], [7], [8]].

Zinc (Zn) is an essential element for body growth, gut and brain development, normal neurological and cognitive functions and immune functions [8,9]. Zn is an antioxidant, heavy metal detoxificant, neurotransmitter (NT) and neuromodulator in the brain [2]. Plasma Zn reflects hippocampus and cerebral cortex Zn concentrations [10,11]. Dietary deficiency, metabolic disturbances, intestinal diseases and immune dysregulations are the possible causes of Zn deficiency [2]. Zn deficiency disrupts the immune response and increases pro-inflammatory cytokines leads to recurrent infection [9]. Intracellular Zn deficiency may cause DNA damage by oxidative stress [7]. During the neurodevelopmental period, Zn deficiency affects both structural and functional development of the brain, causing neuropsychological changes and learning disability [2]. Mutations in genes like zinc transporter 5, SHANK2 and SHANK3 were found in ASD children as a cause of Zn deficiency in them [11]. Researchers also found specific symptomatic improvement in ASD children or in animal models after supplementation with Zn or Zn and vitamin B-6 [7,[12], [13], [14]].

Copper (Cu) is transported by ceruloplasmin (Cp) in the blood. In the brain, it is necessary for nerve conduction and neurotransmitter (NT) like epinephrine, norepinephrine, dopamine and serotonin synthesis [2,10,15]. Liver mainly regulates Cu excretion through faeces by changing its bile concentration [2]. However, excess Cu is toxic, generates free radicals by the easy inter-conversion of cuprous and cupric states and causes oxidative neuronal injury [2]. Excess Cu also causes an imbalance of excitatory and inhibitory neurotransmitters in the central nervous system (CNS), which causes excitability and hyperactivity [2,10,15].

Zn and Cu, as chelating agents bind to and regulate the synthesis of metallothionein (MT) proteins, reducing the body's toxic metal burden. But excess Cu induces MT more potently, prevents systemic and CNS Zn absorption and vice versa, inducing more oxidative stress [2]. Zn and Cu keep balance in blood as cellular Zn uptake and Cp production in liver are enhanced by same cytokines that may reflect Zn deficiency and or toxic Cu status in the body [2].

Cp is a major intrinsic antioxidant protein, involved in the maintenance of Cu homeostasis [16]. It binds plasma Cu reversibly, rendering it non-toxic [2]. Cp acts as ferroxidase and superoxide dismutase, inhibiting the lipid peroxidation of membranes catalyzed by Cu and iron [17]. Within the CNS, Cp is present in astrocytes of the microvasculature, surrounding dopaminergic neurons in the substantia nigra [16]. Increased hepatic Cu pool or free radical injury causes Cp synthesis in the liver, brain and other tissues [2,17]. But excess Cp also decreases both brain serotonin levels and feroxidase activity, altering the ratio of halo to apoceruloplasmin levels and decreasing its half-life [2,16,18].

Available literature suggests strong geographical variations of Zn and Cu levels among ASD children in different countries [8,19] and a close link with Zn/Cu ratio [3,20] and Cp levels [15,21]. Bangladesh is plagued by maternal and child Zn deficiency, as well as rising ASD incidence [5,22]. Unfortunately, only one study was found in South Asia regarding Zn and Cu in ASD children's hair samples in Chennai, India [23]. Few studies have been conducted worldwide to investigate the link between Cp and ASD [15,21]. Therefore, it would be interesting to know the blood levels of these elements in Bangladeshi ASD children. Accordingly, the aim of this study was to determine whether there were any differences in the plasma levels of Zn, Cu, the Zn/Cu ratio, and serum Cp between Bangladeshi ASD children and those of apparently healthy children. The research was also conducted with the aim of using the findings as a potential modifier to cause autism spectrum disorder in Bangladesh in the future.

## 2 Methodology

From July 2018 to December 2018, this cross-sectional comparative study was done at the Department of Physiology, Bangabandhu Sheikh Mujib Medical University (BSMMU), Shahbagh, Dhaka. The Institutional Review Board at BSMMU approved this study's protocol on July 21, 2018, based on the ethical rules of the World Medical Association's Helsinki Declaration.

### 2.1 Study population

A total of 67 subjects were recruited based on effect size [20]. For this purpose, initially, 49 ASD children with an age range of 2–9 years were included from the Parents Forum for Differently Able Children, Mohakhali, Dhaka. The children were already diagnosed by pediatric neurologists in different public and private hospitals and assessed through the Diagnostic and Statistical Manual of Mental Disorders (DSM-5) [1] by certified psychologists. Among the ASD children, 35 subjects were selected finally through purposive sampling as the study group. The control group consists of 32 age, sex and BMI matched apparently healthy children, selected on the basis of selection criteria from the outpatient department, BSMMU.

### 2.2 Study procedure, sample collection and preparation

The subjects' parents were informed of the study's goals and method after selection. Informed written consent was obtained from all voluntarily participating parents, allowing them the freedom to withdraw their children from the study whenever they felt. Following consent taking, the children's personal and dietary histories, as well as their parents' socio-economic, educational and occupational histories were recorded in a data schedule. Thorough physical examinations of the subjects were performed. Exclusion criteria for both ASD and control subjects were children with any gastrointestinal symptoms, acute illness or trauma in the last month, children with hypoalbuminaemia, children with a history of nutritional supplementation in the last two months and children with chelation therapy, chronic liver disease or renal insufficiency. We also did not include children with ASD who had been diagnosed with chromosomal abnormalities and were taking anticonvulsant or antipsychotic drugs. Then, in fasting condition, 5 ml of venous blood was collected from each subject in both groups using a pair of powder free gloves and placed in a metal-free, dry, clean vacutainer. After separating the plasma, it was taken into an eppendrof tube and refrigerated at −80^*◦*^C for the estimation of plasma Zn and Cu in the Department of Immunology, Nutrition and Toxicology Laboratory, International Centre for Diarrhoeal Disease Research, Bangladesh, by the flame atomic absorption spectrophotometry method (Shimadzu AA-6501S, Kyoto, Japan). Then the Zn/Cu ratios were calculated. Complete blood count (CBC), ESR, serum albumin, serum alanine aminotransferase (ALT), serum creatinine levels were measured in the Department of Biochemistry and Molecular Biology, BSMMU (Supplementary Data Table 1). Serum Cp levels were measured in the same department by the immune turbidimetric method, by an automated analyzer (Architect ci8000 system). In this study, Zn deficiency was considered when the plasma level was <0.65 mg/L in the age group 2–5 and <0.78 mg/L in the age group 6–9 [24]. Cu toxicity was defined as plasma level >1.52 mg/L in children aged 2–5 years and >1.33 mg/L in children aged 6–9 years [24].

**Table 1:** Socio-demographic characteristics of ASD and healthy control children.

Demographic Characteristics	Control (n=32)	ASD (n=35)	p-value
Age (Years)^*a*^	5*.*87 *±* 0*.*38 (2 − 9)	5*.*65 *±* 0*.*33 (2 − 9)	0.672^*α*^
Male children [n (%)]	22 (68.75%)	27 (77.19%)	0.582^*β*^
BMI (Kg/m^2^)^*a*^	15*.*57 *±* 0*.*48 (12.77–19.56)	15*.*43 *±* 0*.*45 (12.75–24.32)	0.835^*α*^
**Per capita monthly family income**			
150 dollar-200 dollar	10 (31.2%)	14 (40%)	0.456^*β*^
201 dollar-250 dollar	22 (68.8%)	21 (60%)	
**Educational history of the mother**			
Graduate	23 (71.9%)	24 (68.6%)	0.768^*β*^
Under-graduate	9 (28.1%)	11 (31.4%)	
**Mother's occupation during conception**			
Household activities	8 (25%)	19 (54.3%)	0.015^*β*^
Service	24 (75%)	16 (45.7%)	

^a^Data are expressed as mean ± SE (range) and percentage (%).

^α^Statistical analysis was done by independent sample *t*-test and ^β^Chi square test. n=number of children in each group; ASD = Autism Spectrum Disorder; BMI=Body Mass Index.

### 2.3 Statistical analysis

Data are expressed as mean ± SE, range and percentage. Normal distribution of data was confirmed by the Shapiro-Wilk test. Unpaired t-tests were done to compare the study group and control group. Chi-square tests were done to evaluate the association between each of the variables (Zn deficiency, Cu toxicity) and ASD. Linear regression analysis was done to predict the association between plasma Cu and serum Cp levels. Statistical analysis was done using Statistical Packages for Social Science (SPSS) version 22. During the interpretation of the results, p-values of *≤* 0.05 were considered statistically significant.

## 3 Results

For this study, a total of 67 subjects of both sexes were selected on the basis of inclusion and exclusion criteria. With an age range of 2–9 years, the study group consisted of 35 physician-diagnosed ASD children, and the control group consisted of 32 apparently healthy children. All the biochemical values in both groups were characterized by a Gaussian distribution. In the study population, 77.19% were male. Both groups were matched for age, sex and BMI. There were no statistically significant differences in per capita monthly family income and educational level of the mother between ASD and control children (p=0.456 and p=0.768 respectively). The socio-demographic characteristics of the subjects were summarized in Table 1.

**Fig. 1:**
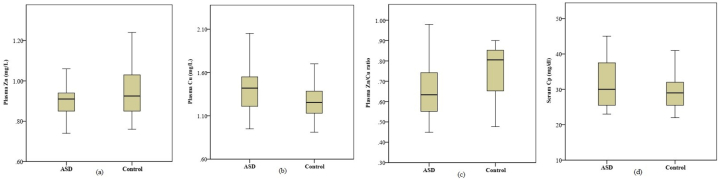
Boxplot of (a) plasma Zn levels, (b) plasma Cu levels,(c) plasma Zn/Cu ratio and (d) serum Cp levels between ASD and healthy control children of 2–9 year age group; total number of children = 67.

Among the 2–9 years old group, the mean plasma Zn levels were significantly lower in the study group than those of the control group (0.89 ± 0.01 mg/L vs. 0.94 ± 0.02 mg/L, p=0.032) (Fig. 1a),. The mean plasma Cu levels were significantly higher in the study group in comparison to the control group (1.40 ± 0.04 mg/L vs. 1.27 ± 0.03 mg/L, p=0.020), (Fig. 1b). Accordingly, mean Zn/Cu ratios were significantly lower in the study group in comparison to the control group (0.66 ± 0.02 vs. 0.76 ± 0.02, p=0.002), (Fig. 1c). The mean serum Cp levels were also significantly higher in the study group in comparison to the control group (31.10 ± 1.20 mg/dl vs. 29.05 ± 0.79 mg/dl, p=0.045), as shown in Fig. 1d.

**Fig. 2:**
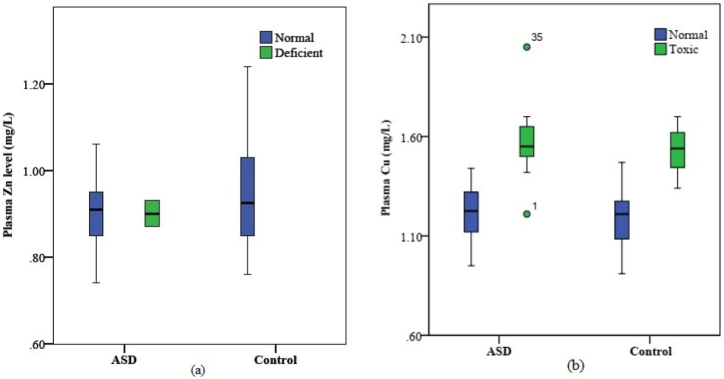
Box plot of association of (a) plasma Zn deficiency and (b) Cu toxicity with ASD in 2–9 years age group children; total number of children = 67.

Analyses performed using the *χ*^2^-test revealed that Zn deficiency was found in 5.71% of ASD children but not in healthy control children. Cu toxicity was found in 49% of ASD children and in 25% of healthy control children. Both Zn deficiency and Cu toxicity were significantly associated with ASD compared to healthy controls (*χ*^2^=59.239, p=0.000 and *χ*^2^=4.313, p=0.05, respectively), Fig. 2a, b.

According to the laboratory normal reference values of plasma Zn and Cu, all the subjects in both groups were divided into two age groups: 2–5 years and 6–9 years. The differences in age between the ASD and control groups were statistically non-significant (p=0.714). Therefore, both groups were matched for age (Table 2).

**Table 2:** Distribution of the subjects by age in ASD children and healthy control children aged 2 to 9 years.

	Age Group		Total (n)	*χ*^2^-test	p-value	
	2–5 years	6–9 years	67			
ASD^*a*^	17 (48.6%)	18 (51.4%)	35	0.134		0.714^ns^
Control^*a*^	18 (56.2%)	14 (43.8%)	32			

^ns^—non-significant (p 0.05).

^a^Data are expressed as number of subjects (n) and as percentage (%). Statistical analysis was done by Chi–Square test to compare between groups.

Considering the age group, 2–5 years old ASD children showed a significant difference in plasma Cu and Zn/Cu ratios. The mean plasma Cu levels were significantly higher (1.42 ± 0.06 vs. 1.20 ± 0.05, p=0.009) and the mean Zn/Cu ratios were significantly lower (0.65 ± 0.03 vs. 0.81 ± 0.02, p=0.001) in ASD children than that of control. There were no significant differences in mean plasma Zn and serum Cp levels between the study and control children of the same age group (Table 3). In the age group 6–9 years, no significant differences were observed for plasma Zn (p=0.166), Cu (p=0.590), Zn/Cu ratios (p=0.590) and serum Cp (p=0.673) levels (Table 4).

**Table 3:** Plasma Zn, Cu, Zn/Cu ratio and serum Cp levels of the ASD and healthy control children of 2–5 years age group.

Biochemical Parameters	ASD (n=17)	Control (n=18)	*t*-Test	p-value
Plasma Zn (mg/L)	0.90 ± 0.02 (0.74–1.06)	0.96 ± 0.03 (0.76–1.24)	−1.603	0.119
Plasma Cu (mg/L)	1.42 ± 0.06 (1.06–2.05)	1.20 ± 0.05 (0.91–1.70)	2.780	0.009*
Plasma Zn/Cu	0.65 ± 0.03 (0.45–0.95)	0.81 ± 0.02 (0.48–0.90)	−3.507	0.001*
Serum Cp (mg/dl)	30.19 ± 1.61 (23–42)	27.15 ± 0.86 (22–34)	1.661	0.110

Data are expressed as mean ± SE (range). Statistical analysis was done by independent sample *t*-test. n=number of children in each group; *=statistically significant (p≤ 0.05).

**Table 4:** Plasma Zn, Cu, Zn/Cu ratio and serum Cp levels of the ASD and healthy control children of 6–9 years age group.

Biochemical Parameters	ASD (n=18)	Control (n=14)	*t*-Test	p-value
Plasma Zn (mg/L)	0.89 ± 0.02 (0.74–1.03)	0.93 ± 0.02 (0.79–1.05)	−1.425	0.166 ^ns^
Plasma Cu (mg/L)	1.38 ± 0.05 (0.95–1.70)	1.34 ± 0.04 (1.06–1.58)	0.545	0.590 ^ns^
Plasma Zn/Cu	0.66 ± 0.03 (0.48–0.98)	0.70 ± 0.03 (0.54–0.86)	−0.836	0.590 ^ns^
Serum Cp (mg/dl)	33.13 ± 1.57 (23–42)	32.21 ± 1.49 (23–45)	0.426	0.673 ^ns^

Data are expressed as mean ± SE (range). Statistical analysis was done by an independent sample *t*-test. n=number of children in each group; ^ns^ = Non-significant (p≤0.05).

Toxic plasma Cu levels were significantly associated with ASD in 2–5 years olds (p=0.011). No significant association was observed between the toxic plasma Cu level and ASD in the 6–9 years age group (p=0.724), (Table 5).

**Table 5:** Association of toxic plasma Cu levels with ASD in children of different age groups.

		Plasma Cu level		Total (n)	p-value
		Normal	Toxic		
Age 2–5 years	ASD	11 (64.7%)	6 (35.3%)	17	0.011*
	Control	16 (88.9%)	2 (11.1%)	18	
Age 6–9 years	ASD	8 (44.4%)	10 (55.6%)	18	0.724 ^ns^
	Control	7 (50%)	7 (50%)	14	

The data are expressed as the number of subjects. Figures in parentheses indicate percentages. Statistical analyses were done by the Chi-Square test. ^ns^=Non-significant (p>0.05); *: Statistically significant (p ≤ 0.05); n=number of subjects.

**Fig. 3:**
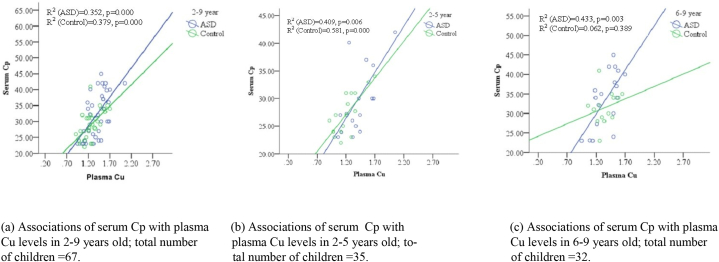
Associations of serum Cp with plasma Cu levels in different age groups (a) 2–9 years (b) 2–5 years (c) 6–9 years. Regression analysis (R squared) was performed as the test of significance. ASD children-study group; Statistically significant (P≤0.05).

To predict the increment of Cp as an antioxidant due to the increase of Cu, linear regression tests were done in all the age groups of ASD and control children. Results showed that a 35.2% increment in Cp can be accounted for by the increase of Cu in the 2–9 years age group of ASD children (F (1, 33) = 17.918, p<0.001) versus 37.9% increment in Cp can be accounted for by the increment of Cu in the same age control children (F (1, 30) = 18.293, p<0.001). (Table 6, Fig. 3a). In the 2–5 years old ASD children group, 40.9% of the increment in Cp can be accounted for by the increment of Cu (F (1, 15) = 10.381, p<0.05) versus 58.1% increment in Cp can be accounted for by the increment of Cu in control children of the same age (F (1, 12) = 22.152, p<0.0). (Table 6, Fig. 3b). In 6–9 years old ASD children, 43.3% increment in Cp can be accounted for by the increment of Cu (F (1, 16) = 12.212, p<0.003). Whereas, in same-age control children, only 6.2% increment in Cp can be accounted for by the increment of Cu (F (1, 16) = 0.799, p>0.05). (Table 6, Fig. 3c).

**Table 6:** Regression analysis of plasma Cu and serum Cp in ASD and control children of 2–9, 2–5 and 6–9 years age group.

		Standardized Coefficients Beta	R^2^	t-value	df	F	p-value
2–9 years	ASD (n = 35)	.593	.352	4.233	1, 33	17.918	.000*
	Control (n = 32)	.615	.379	4.277	1, 30	18.293	.000*
2–5 years	ASD (n = 17)	.640	.409	3.222	1, 15	10.381	.006*
	Control (n = 18)	.762	.581	4.707	1, 16	22.152	.000*
6–9 years	ASD (n = 18)	.658	.433	3.495	1, 16	12.212	.003*
	Control (n = 14)	.250	.062	.894	1, 12	.799	.389

Predictors: (Constant); Cu, Dependent Variable: Cp; ASD children-study group; n=number of subjects *: Statistically significant (p≤0.05).

## 4 Discussion

This study attempted to determine whether there was a significant difference in the blood levels of Zn, Cu, the Zn/Cu ratio and Cp between Bangladeshi ASD children and children who were apparently healthy. A number of investigators have proposed a variety of hypotheses on the implications of these metals throughout the neurodevelopmental period as well as in the later lives of ASD children [9,19].

In this particular investigation, the mean plasma Zn level of the study group was significantly lower than that of the control group in children who were between the ages of 2 and 9 years old. In various blood samples, researchers in Egypt [25], Turkey [26], the United Kingdom [9], China [20] and Romania [3] came to almost identical conclusions, which were in line with the findings of the current study. However, despite the fact that the mean Zn level in children with ASD was lower than in healthy children, this study did not identify any statistically significant differences in the mean Zn levels between the study group and the control group in any of the age groups ranging from 2 to 5 years or from 6 to 9 years. Studies conducted in a variety of states in the United States of America also observed comparable results in plasma, serum, or hair samples taken from children with ASD spanning a range of ages, from early infancy to adulthood [8,10,27,28].

According to the findings of this study, among all the ASD children (ages between 2 and 9 years), 5.71% had Zn deficiency. Zn deficiency was also found significantly associated (p = 0.000) with ASD when compared to that of healthy controls. Zn insufficiency was identified in roughly 10% of very similarly aged ASD children in a cohort of 28 Romanian children, compared to that of healthy control children [3]. A greater prevalence of Zn deficiency was also discovered by Goyal DK et al., 2019 among children with ASD who were under the age of 16, living in Manchester, UK; however, the difference in mean blood Zn levels between the ASD group and the healthy control group was not statistically significant [9]. In Japan, a large cohort of 1,967 children with ASD aged 0–3 years old had Zn deficiency at a rate of 29.7% in hair samples [6].

In this particular investigation, the mean plasma Cu level in the study group was significantly higher compared to the control group in the age groups ranging from 2 to 9 years and 2–5 years respectively. In the United States of America, researchers noticed almost the same results in plasma or serum samples of ASD children who were in a fairly comparable age range [10,15,20]. But ASD children in Romania and Brazil did not find a significant difference in their plasma or whole blood Cu levels when compared to children from the control group [3,29].

On the other hand, this research revealed an incidence of Cu toxicity that was 24% higher in children with ASD who were in the 2–9 years age range compared to that of healthy controls. Cu toxicity was found to have a significant association with ASD in children aged 2–9 years as well as those aged 2–5 years.

Accordingly, the results of this study showed that the ratio of Zn to Cu in the study group was significantly lower than that of the control group in the age ranges of 2–9 years and 2–5 years, but not in the age range of 6–9 years. Li S.-o et al., 2014 found almost similar serum findings in Chinese ASD children [20]. El-Meshad GM et al., 2017 also found similar findings for plasma Zn and serum Cu levels of Egyptian ASD children [25]. Interestingly, a study in the neighboring country of India also found elevated mean Cu levels in hair samples of 4–12 years old low, medium, and high-functioning ASD children. On the other hand, Zn deficiency was only detected in low-functioning ASD children [23]. But in Romania and in Illinois, USA, the Zn/Cu ratio was decreased either due to an increase in whole blood Cu levels or due to decreased plasma Zn levels [3,10]. A decreased Zn/Cu ratio in a hair sample due to a lower mean Zn level and a higher mean Cu level was also observed in another cohort of Indian ADHD children than that of healthy control children [30].

Toxic Cu directly damages neurons by unbalancing the antioxidant system [31]. It causes Zn deficiency, dysregulates dopamine, serotonin and epinephrine levels, interferes with adrenal hormone production, de-creases Zn absorption in pre- and postsynaptic neurons, and decreases the conversion of glutamate to gamma amino butyric acid (GABA) [26]. Copper is also an inhibitor of GABA-mediated responses, especially in Purkinje cells and causes neuronal excitation in ASD children [10]. Scientists suggested that Zn deficiency and Cu toxicity further affect each other's concentrations by decreasing the expression of Zn uptake *trans*porters in enterocytes and the CNS [32], and by MT dysregulation [2]. Both mechanisms cause excess Cu or other toxic heavy metal absorption in the GIT and in brain neurons [2]. MT dysfunction, Zn deficiency and Cu toxicity are all responsible for neurotoxicity by impairing the antioxidant defenses [3]. Both Zn deficiency and Cu toxicity also have implications for the development of other neurological diseases like Alzheimer's and Parkinson's diseases [3]. Therefore, in this present study, a lower Zn/Cu ratio in ASD children may reflect a generalized low Zn concentration and high Cu levels in plasma with decreased efficiency of the antioxidant system.

Though this study observed an increasing trend in mean Cp levels in all three age groups of ASD children, a significantly higher mean level was found only in the 2–9 years age group than that of the control group. El-Baz F et al., 2018 also found higher serum Cp levels in ASD children [15]. Whereas, some scientists reported a significantly lower level of plasma Cp in ASD children in comparison to their healthy siblings [21]. According to the findings of the regression analysis, Cp had a significant association with variations in Cu in almost all age groups of ASD and control children, with the exception of children aged 6–9 years old who were in the control group. It is important to note that although only 11.1% of control children aged 2 to 5 years old were found to have toxic levels of Cu in their plasma, the Cp had a relatively high R^2^ value for Cu, which was 0.581. (Table 5, Table 6). In contrast, children with ASD of the same age had a moderate R^2^ value for plasma Cu (0.409), despite the fact that 35.3% of them had Cu toxicity. The R^2^ value of serum Cp for plasma Cu in ASD children aged 2–9 and 6–9 were moderate (0.352 and 0.433), as was the R^2^ value of the controls, who were 2–9 years old (0.379) (Table 6). In this study, Cp might be increased in ASD children due to an increased hepatic Cu pool and by free radical injury in the brain [2,17]. Scientists suggested that, toxic Cu induced reactive oxygen species impairs Cu binding with Cp. As a result, Cp feroxidase activity is reduced, which alters the ratio of halo to apoceruloplasmin levels, decreases its half-life and makes more Cu available to promote oxidative injury [16,17]. Researchers also found that, in the brain, both toxic Cu and excess Cp decrease serotonin levels by inhibiting the enzyme hydroxytryptophan decarboxylase; thus increasing neuronal excitation [10,23].

This study was conducted in Bangladesh, which is struggling against poverty, where ASD imposes an additional financial burden on the government to meet the Sustainable Development Goals by 2030 [22]. One of the limitations of this study was its small sample size (35 ASD children and 32 control children). We also did not correlate the metal levels with the severity of ASD and did not measure the Cp antioxidant capacity. The study's strength, however, was in establishing the association between plasma Zn deficiency and Cu toxicity and ASD. Since Cp is a potential marker in diseases associated with Cu, this research also found an association between serum Cp levels and plasma Cu levels in these particular ASD children.

## 5 Conclusions

From this study, it is revealed that ASD is associated with Zn deficiency, Cu toxicity and higher Cp levels. Low plasma Zn and/or high plasma Cu levels consequently lower the Zn/Cu ratio. This may be indicative of inadequate CNS functions and neurotoxicity in ASD children. Moreover, excess Cp-induced neuronal excitability may also have contributed to the development of ASD. Further research is needed to explore the association of Zn, Cu, Zn/Cu ratio and Cp with ASD in Bangladesh.

## Author contribution statement

Umme Raihan Siddiqi: Conceived and designed the experiments; Performed the experiments; Analyzed and interpreted the data; Contributed reagents, materials, analysis tools or data; Wrote the paper.

Shelina Begum: Conceived and designed the experiments.

Shorifa Shahjadi: Conceived and designed the experiments; Contributed reagents, materials, analysis tools or data.

Sharmin Afroz; Syeda Nusrat Mahruba; Jobaida Parvin; Md. Mahbubur Rahman: Contributed reagents, materials, analysis tools or data.

## Data availability statement

Data will be available upon reasonable request.

## Declaration of competing interest

The authors declare that they have no known competing financial interests or personal relationships that could have appeared to influence the work reported in this paper.
