# Supplementary material for: Plasma zinc, copper and serum ceruloplasmin levels of autism spectrum disorder children in Bangladesh
Source: Heliyon. 2023 Aug 9;9(8):e18624. doi: 10.1016/j.heliyon.2023.e18624 (PMC10448426; doi:10.1016/j.heliyon.2023.e18624)
Supplement: Multimedia component 1 [file mmc1.docx]

# Supplementary Data

Table 1: Baseline data of the ASD and healthy control children (N=67)

| Biochemical Parameters | ASD (n= 35) | Control (n= 32) | t-Test | p-value |
| --- | --- | --- | --- | --- |
| Hemoglobin (gm/dl) | 12.16 ± 0.17 (9.50-13.80) | 12.25 ± 0.21 (10.00-14.80) | -.344 | 0.732 ^ns^ |
| Total WBC Count(X10^12/L) | 9.09±0.3 (4.70-11.00) | 8.13± 0.41 (4.50-11.00) | 1.88 | 0.06 ^ns^ |
| Total RBC Count (X10^9/L) | 4.75± .06 (3.69-5.30) | 4.84 ± 0.06 (4.41-5.48) | -1.004 | 0.319 ^ns^ |
| T Platelet Count (X10^9/L) | 314.34 ±12.77 (150-455) | 301.09 ± 13.35 (180-500) | 0.717 | 0.476 ^ns^ |
| Serum Albumin (gm/L) | 4.41±0.064 (3.80-5.00) | 4.42±0.063 (4.00-5.20) | -0.079 | 0.938 ^ns^ |
| Serum Creatinine (mg/dl) | 0.50±0.027 (0.28-1.29) | 0.48±0.026 (0.06-0.70) | 0.338 | 0.736 ^ns^ |
| ALT(U/L) | 18.08± 0.98 (12-39) | 17.9± 0.8 (12-32) | 0.767 | 0.446 ^ns^ |

Data are expressed as mean *±* SE (range). Statistical analysis was done by independent sample t-test. ASD children- study group; N= total number of children; n= number of children in each group; ^ns^- Non-significant (p>0.05).
